# Catheter-Associated Vancomycin-Resistant Enterococcus faecium Ventriculitis and Multidrug-Resistant Acinetobacter baumannii Pneumonia With Subsequent Acinetobacter Ventriculitis: A Case Report

**DOI:** 10.7759/cureus.49058

**Published:** 2023-11-19

**Authors:** Kyle M Rei, Vedhika Reddy, Christopher Andraos, James Brazdzionis, Javed Siddiqi

**Affiliations:** 1 Neurosurgery, California University of Science and Medicine, Colton, USA; 2 Neurosurgery, Arrowhead Regional Medical Center, Colton, USA; 3 Neurosurgery, Riverside University Health System Medical Center, Moreno Valley, USA; 4 Neurosurgery, Desert Regional Medical Center, Palm Springs, USA

**Keywords:** ventriculostomy, intrathecal, antibiotic therapy, cerebrospinal fluid shunt, pneumonia, multidrug resistant (mdr), ventriculitis, acinetobacter baumanii, enterococcus faecium, vancomycin resistant enterococcus (vre)

## Abstract

Ventriculitis is associated with cerebrospinal fluid (CSF) shunts, and rare microorganisms associated with infection include vancomycin-resistant *Enterococcus* (VRE) *faecium* and *Acinetobacter baumannii*. Both organisms are known to cause nosocomial infections, and the emergence of multidrug-resistant (MDR) strains presents a treatment challenge. There is a lack of consensus on antimicrobial agent selection for ventriculitis involving VRE *faecium* or MDR *A. baumannii*, which are life-threatening conditions. We present a case of a 59-year-old male presenting with CSF catheter-associated VRE *faecium* ventriculitis and MDR *A. baumannii* pneumonia who subsequently developed a nosocomial MDR *A. baumannii *ventriculitis. Both instances of ventriculitis were successfully treated with combination antibiotic therapy. VRE *faecium* ventriculitis was successfully treated with linezolid and intrathecal daptomycin. While daptomycin is not approved for *Enterococcal* infections, the synergistic effect of daptomycin in combination with linezolid proved effective. Although the MDR *A. baumannii* pneumonia was not cured with cefiderocol monotherapy, the MDR *A. baumannii* ventriculitis was successfully treated with combination therapy including cefiderocol, ampicillin/sulbactam, and intrathecal colistin. This highlights life-saving combination antibiotic therapies for ventriculitis caused by multiple rare and drug-resistant microorganisms.

## Introduction

Ventriculitis is the inflammation of the ependymal lining of the cerebral ventricles and is also known as ependymitis, ventricular empyema, pyocephalus, and pyogenic ventriculitis [1]. Neurosurgical patients with cerebrospinal fluid (CSF) shunts are at increased risk of developing ventriculitis, and the four mechanisms of CSF shunt infection include (1) colonization during placement, (2) retrograde infection from the distal end (e.g., ventriculoperitoneal (VP) shunt perforating the bowel with enteric bacteria ascending the shunt), (3) infection through the skin after needle insertion, and (4) hematogenous seeding (e.g., ventriculoatrial shunt increasing the risk of developing bacteremia, in which bacteria could ascend the shunt) [2]. The most common organisms associated with CSF shunt infection are coagulase-negative staphylococci (especially *Staphylococcus* epidermidis), *S. aureus*, *Propionibacterium acnes*, and Gram-negative bacilli [2].

Enterococcus is a rare cause of ventriculitis that has been associated with CSF shunts, particularly VP shunts, likely due to the proximity of the bowel and the potential for ascending infection [3,4]. Enterococci are Gram-positive bacteria found in the normal bowel microbiota [3]. The species most commonly responsible for infection include *Enterococcus faecalis* and *E. faecium*, which account for over 90% of clinical isolates, and less commonly *E. gallinarum* [4]. Accepted as the leading cause of nosocomial infections, these bacteria are associated with bacteremia, urinary tract infections, endocarditis, biliary tract infections, and rarely central nervous system (CNS) infections [5]. Vancomycin-resistant Enterococcus (VRE) strains present a treatment challenge, and 30% of healthcare-associated Enterococcus infections in 2017 were resistant to vancomycin [6].

*Acinetobacter baumannii* is a rare cause of ventriculitis that has been associated with invasive procedures and the use of devices, such as CSF shunts, secondary to contamination [7,8]. Procedures involving suction and pulsatile lavage have been identified as high-risk activities that may lead to environmental contamination [9]. *A. baumannii* is a Gram-negative rod-shaped bacterium that is an emerging nosocomial pathogen and is often multidrug-resistant (MDR) [7,10]. In addition to being ubiquitous in the environment, Acinetobacter is often found on human skin and mucous membranes [10]. These bacteria have been associated with bacteremia, pneumonia, meningitis, urinary tract infections, and surgical wound infections [11]. Acinetobacter contains up to 45 resistance genes, leading to the emergence of strains resistant to most commercially available antibiotics [8].

The prudent selection of antimicrobial agents to treat these rare and life-threatening CNS infections is of great significance. There is a lack of consensus for the optimal treatment of VRE CNS infections, and although carbapenems have been the standard for empirical treatment of MDR-*A. baumannii*, the emergence of carbapenem-resistant strains indicates a need for alternative therapy [6,12].

We present the case of a 59-year-old male presenting with CSF catheter-associated VRE faecium ventriculitis and MDR-*A. baumannii* pneumonia who subsequently developed a nosocomial MDR-*A. baumannii* ventriculitis. Both instances of ventriculitis were successfully treated with combination antibiotic therapy. Approval was obtained from the IRB in connection with Arrowhead Regional Medical Center for this case study.

## Case presentation

A 59-year-old male with a history of trigeminal neuralgia treated with microvascular decompression four months prior and subsequent external ventricular drain (EVD) placement, emergent suboccipital craniectomy with hematoma evacuation, VP shunt placement, tracheostomy, and percutaneous endoscopic gastrostomy (PEG) placement was brought from a rehabilitation facility to the emergency department due to intermittent fevers lasting three weeks with known pneumonia and pseudomeningocele in the left suboccipital region without a work-up being completed at the rehabilitation facility.

**Figure 1:**
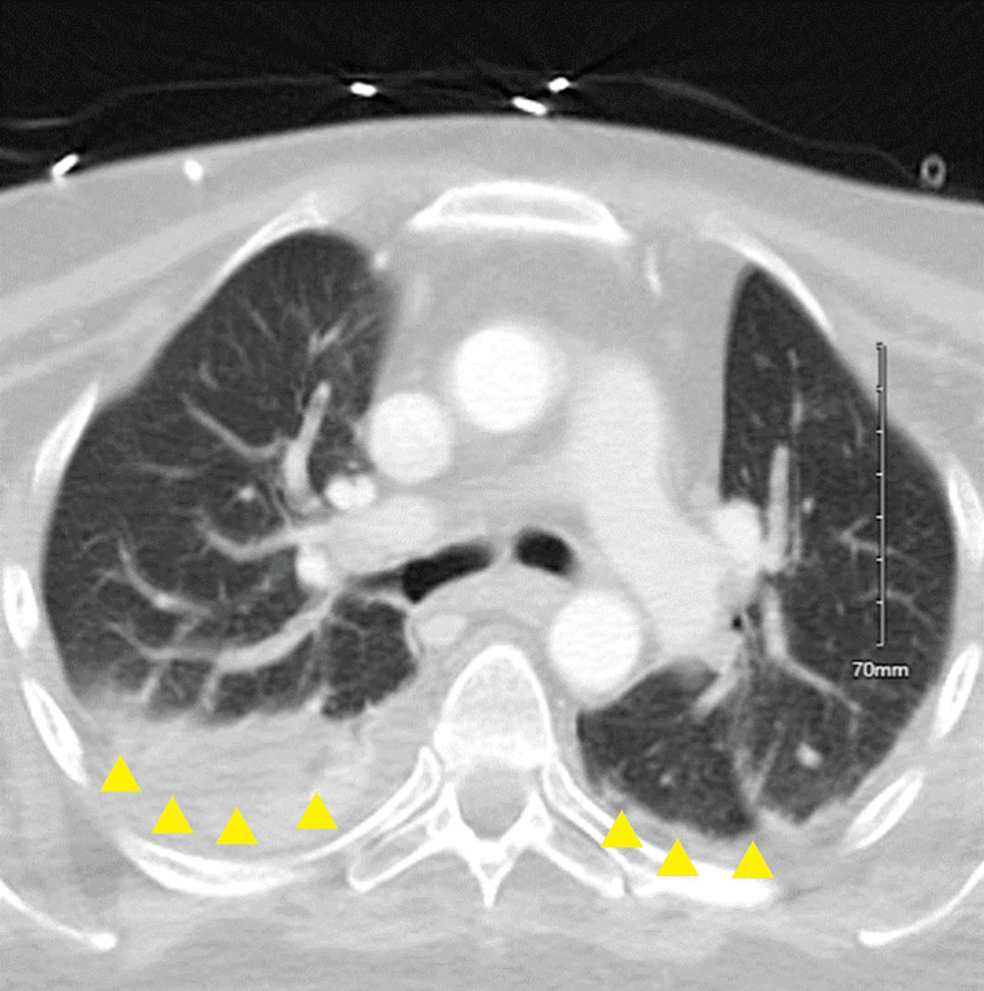
CT chest with IV contrast day 1 CT chest with IV contrast imaged on hospitalization day 1 with bilateral pleural effusion right worse than left. Bilateral lower lung airspace disease was identified.

Upon admission, CT chest with contrast showed evidence of bilateral lower lung airspace disease, and sputum culture yielded MDR-*A. baumannii* with many Gram-positive and Gram-negative bacilli (Figure 1). A blood culture taken on admission yielded no growth, and a CSF culture yielded growth of VRE faecium (Table 1). The VP shunt was tapped on admission, and CSF analysis found a colorless, hazy specimen with a WBC count of 395.0 cells/µL (72% neutrophils, 6% lymphocytes, and 22% macrophages), an RBC count of 66.0 cells/µL, a glucose level of 45 mg/dL, and a protein level of 199 mg/dL (Table 2). The CBC findings are reported in Table 3. Initial therapy included vancomycin, cefepime, and metronidazole. The MDR-*A. baumannii* pneumonia was unsuccessfully treated with an eight-day course of cefiderocol (2 g, IV, q8h). Follow-up respiratory cultures continued to show MDR-*A. baumannii* growth at days 14, 24, and 27.

**Table 1:** Microbiology susceptibility Microbiology susceptibility testing for sputum, CSF, and endotracheal tube (ETT) aspirate samples collected throughout hospitalization. MDR: multidrug-resistant; VRE: vancomycin-resistant Enterococcus.

Day	1	1	31	80	80
Test	Sputum culture	CSF culture	CSF culture	ETT aspirate culture	ETT aspirate culture
Microorganism	MDR-*A. baumannii*	VRE faecium	MDR-*A. baumannii*	MDR-*A. baumannii*	Citrobacter koseri
Amikacin	Resistant	N/A	Resistant	Resistant	Susceptible
Ampicillin	N/A	Resistant	N/A	N/A	Resistant
Ampicillin and sulbactam	Intermediate	N/A	Resistant	Intermediate	Susceptible
Cefazolin	N/A	N/A	N/A	N/A	Susceptible
Cefepime	Resistant	N/A	Resistant	Resistant	Susceptible
Ceftazidime	Resistant	N/A	Intermediate	Intermediate	N/A
Cefuroxime	N/A	N/A	N/A	N/A	Intermediate
Ciprofloxacin	Resistant	N/A	Resistant	Resistant	Susceptible
Gentamicin	Resistant	N/A	Resistant	Resistant	Susceptible
Imipenem	Resistant	N/A	Resistant	Resistant	N/A
Levofloxacin	Resistant	N/A	Resistant	Resistant	Susceptible
Meropenem	Resistant	N/A	Resistant	Resistant	
Piperacillin and tazobactam	N/A	N/A	N/A	N/A	Susceptible
Tetracycline	N/A	N/A	Resistant	N/A	N/A
Tobramycin	Resistant	N/A	Resistant	Resistant	Susceptible
Trimethoprim and sulfamethoxazole	Resistant	N/A	Resistant	Resistant	Susceptible
Vancomycin	N/A	Resistant	N/A	N/A	N/A

**Table 2:** Cerebrospinal fluid analyses CSF analysis results at the time of notable events during hospitalization. MDR: multidrug-resistant; VRE: vancomycin-resistant Enterococcus.

Day	1	25	31	66
Event	CSF culture grows VRE faecium	Completed linezolid and intrathecal daptomycin	CSF culture grows MDR-*A. baumannii*	Nearly completed cefiderocol, ampicillin/sulbactam, and intrathecal colistin
Volume (mL)	6.0	N/A	3.0	2.0
Color	Colorless	Yellow	Straw	Colorless
Appearance	Hazy	Cloudy	Clear	Cloudy
WBC (µL)	395.0	198.0	309.0	3.0
RBC (mm^3^)	66.0	6,000.0	633.0	1,000.0
Glucose (mg/dL)	45	53	5	76
Protein (mg/dL)	199	230	<4	31
Neutrophil (%)	72	85	95	0
Lymphocyte (%)	6	9	2	0
Macrophage (%)	22	6	3	100

**Table 3:** Complete blood count labs CBC results at the time of notable events during hospitalization. MDR: multidrug-resistant; VRE: vancomycin-resistant Enterococcus.

Day	1	25	31	75	84	100
Event	CSF culture grows VRE faecium	Completed linezolid and intrathecal daptomycin	CSF culture grows MDR-*A. baumannii*	Completed cefiderocol, ampicillin/sulbactam, and intrathecal colistin	ETT aspirate culture grows MDR-*A. baumannii* and *C. koseri*	Completed cefiderocol and meropenem
WBC (10^3^/µL)	14.9	12.7	14.5	12.1	15.4	16.2
RBC (10^6^/µL)	2.69	3.73	3.36	3.37	3.66	4.05
Hemoglobin (g/dL)	9.2	12.2	10.7	10.2	11.0	12.4
Neutrophils (%)	73	71	82	68	69	73
Lymphocytes (%)	12	10	8	7	6	7
Monocytes (%)	9	8	7	11	8	6
Eosinophils (%)	4	8	1	8	15	11
Basophils (%)	1	1	1	1	1	1
Platelets (10^3^/µL)	577	438	343	441	470	517

**Figure 2:**
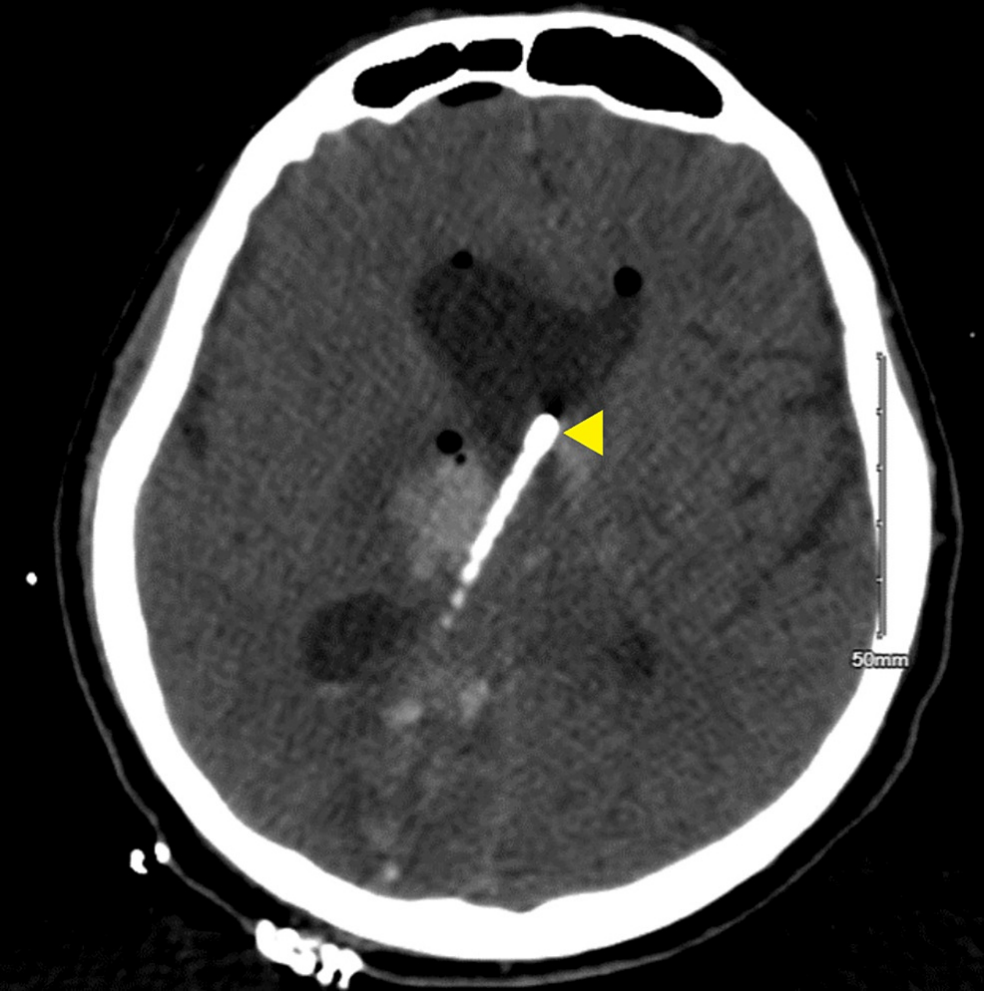
CT head without IV contrast on day 15 showing ventriculostomy placement in right ventricle CT head without IV contrast imaged on hospitalization day 15 showing ventriculostomy placement in the right ventricle for non-communicating hydrocephalus.

**Figure 3:**
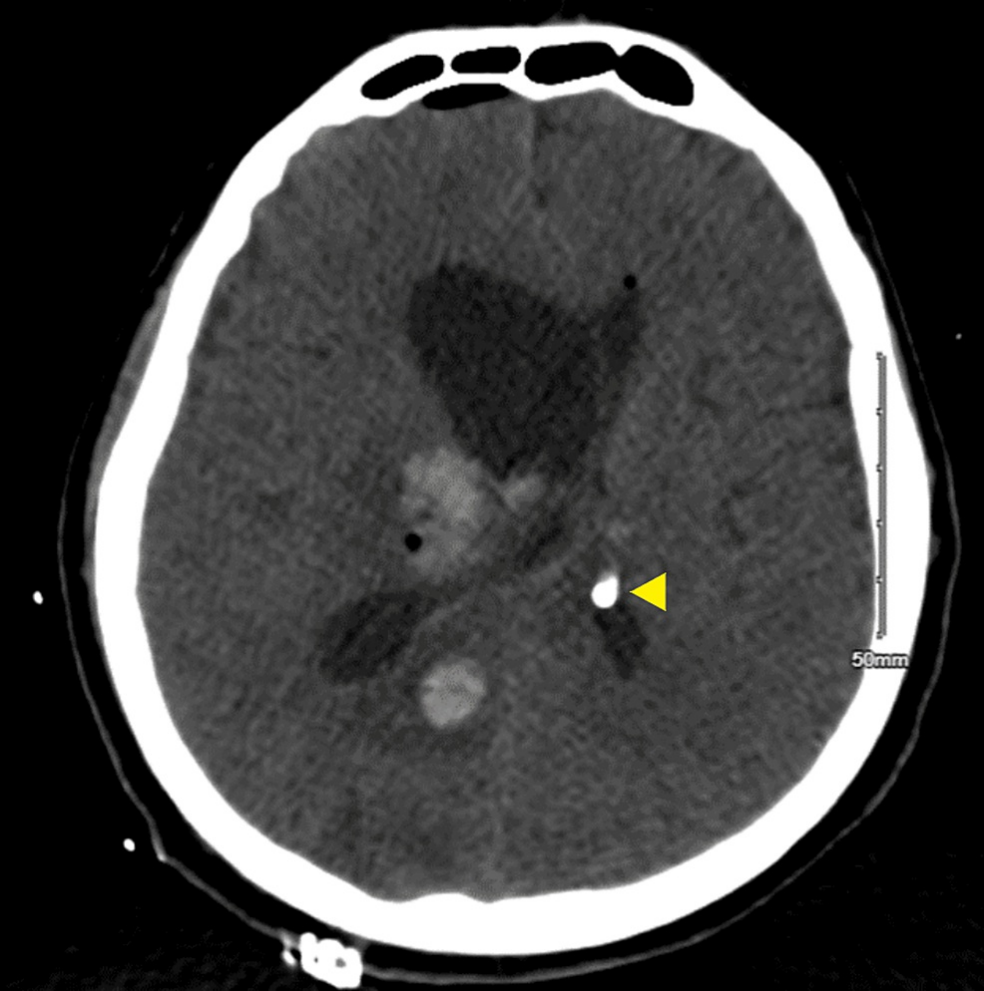
CT head without IV contrast on day 15 showing ventriculostomy placement in left ventricle CT head without IV contrast imaged on hospitalization day 15 showing ventriculostomy placement in the left ventricle for non-communicating hydrocephalus.

**Figure 4:**
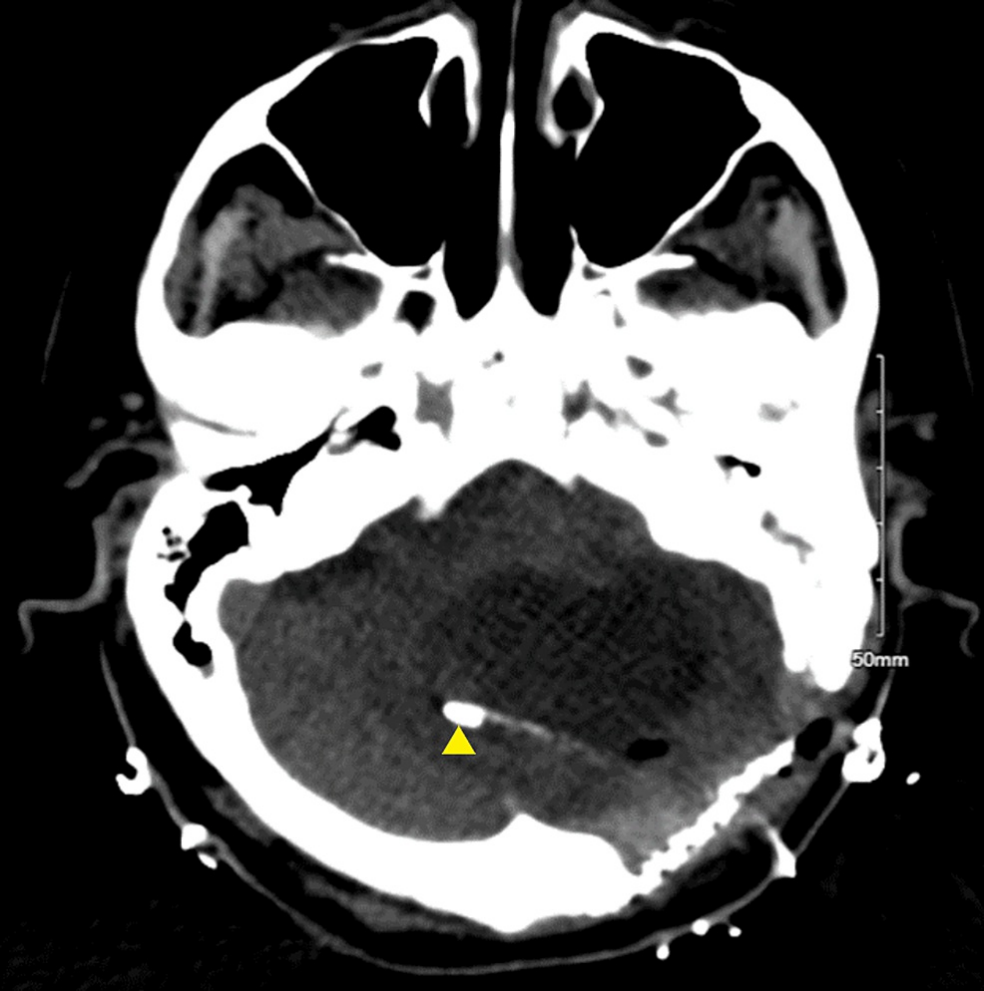
CT head without IV contrast on day 15 showing ventriculostomy placement in fourth ventricle CT head without IV contrast imaged on hospitalization day 15 showing ventriculostomy placement in the fourth ventricle for non-communicating hydrocephalus.

The VRE faecium ventriculitis was treated with a 21-day course of linezolid (600 mg, PEG, q12h) and a 14-day course of intrathecal daptomycin (5 mg, q72h). Additionally, the VP shunt was removed and replaced with a right-frontal EVD. On day 13, this EVD was replaced, and on day 14, two additional EVDs were placed (left lateral and fourth ventricular) due to evidence of trapped lateral ventricles bilaterally and an enlarged fourth ventricle (Figures 2-4). Serial CSF cultures taken every one to three days, beginning on day 4, yielded no growth at this time.

On hospital day 31, CSF culture showed MDR-*A. baumannii* growth, and CSF analysis found a straw-colored, clear specimen with a WBC count of 309.0 cells/µL (95% neutrophils, 2% lymphocytes, and 3% macrophages), an RBC count of 633.0 cells/µL, a glucose level of 5 mg/dL, and a protein level of <4 mg/dL. Five days prior, a blood culture was negative.

The MDR-*A. baumannii* ventriculitis was treated with a 44-day course of cefiderocol (2 g, IV, q8h) and ampicillin/sulbactam (9 g, IV, q8h) and a 14-day course of intrathecal colistin (5 mg, q12h). Additionally, on day 31, the right frontal EVD was removed and the left lateral EVD was replaced due to potential biofilm. The right frontal EVD was then replaced after evidence of non-communicating hydrocephalus on the post-EVD scan. The fourth ventricular EVD was not changed due to a significant decrease in ventricular size and procedural risk. On day 33, CSF culture again yielded MDR-*A. baumannii*, and left, right, and posterior fossa EVDs were replaced. Beginning on day 35, serial CSF cultures collected every one to three days over the next month, including fungal and anaerobic cultures, yielded no growth. On hospital day 69, bilateral VP shunts were placed.

On day 80, the endotracheal tube (ETT) aspirate culture yielded MDR-*A. baumannii* and *Citrobacter koseri*. Blood cultures yielded no growth at this time. A 14-day course of antibiotics was given: cefiderocol (2 g, IV, q8h) for Acinetobacter and meropenem (2 g, IV, q8h) for Citrobacter. After this course of antibiotics, blood culture yielded no growth, but sputum culture continued to show growth of MDR-*A. baumannii* and *C. koster*i. On day 103, the patient was discharged.

## Discussion

The etiology of our patient’s VRE faecium ventriculitis is likely retrograde infection from the peritoneal end of the VP shunt due to the identity of the pathogen, an enteric bacterium. Additionally, due to the absence of radiographic evidence of bowel perforation or blood cultures growing VRE faecium, the mechanism appearing to drive this infection was bacterial translocation (BT). BT has been defined as the movement of viable and non-viable microbes and microbial products from the intestinal lumen through the epithelial mucosa into the mesenteric lymph nodes and possibly other organs [13]. BT is promoted by bacterial overgrowth of a few types of intestinal bacteria, including enterococci, physical disruption of the gut mucosal barrier, and impaired host defense, and BT has been associated with many clinical conditions, including increased intra-abdominal pressure [13,14]. The patient’s presentation with chronic constipation, yet no radiographic evidence of intestinal obstruction, provides a possible underlying etiology for the BT; however, to the best of our knowledge, claiming a definitive etiology is not plausible given the complexity of both the BT and the patient’s presentation.

Our patient’s VRE faecium ventriculitis was successfully treated with linezolid and intrathecal daptomycin. While linezolid and daptomycin have found their place in treating VRE bloodstream infections, there is a lack of consensus for the optimal treatment of VRE CNS infections due to the limitations of each drug: although linezolid has good CNS penetration, linezolid is limited to bacteriostatic activity for Enterococcus species; although daptomycin has bactericidal activity for Enterococcus species, daptomycin has limited CNS penetration due to its large molecular weight and high degree of protein binding [6]. In vitro models have demonstrated synergistic killing of MDR-Enterococcus strains with a combination of linezolid and daptomycin. While the mechanism is unclear, it is hypothesized that daptomycin increases the access of linezolid to its target on the ribosome by penetrating and depolarizing the cell membrane [15]. In addition to the benefit of their synergistic effects, daptomycin monotherapy is cautioned due to the possible development of daptomycin-resistant mutants [16]. The mechanism of this resistance includes genetic changes involving two pathways: (1) LiaFSR, associated with cell envelope stress response, and (2) YycFGHIJ, associated with regulation of cell wall homeostasis [17].

Reviewing the effectiveness of linezolid in treating VRE faecium CNS infections, Lee et al. conducted a literature review of PubMed/MEDLINE case reports and found that 15/19 cases reported clinical cure (78.9%). Among those cured, 8/15 used linezolid monotherapy. The remaining 7/15 cases used linezolid in combination with ampicillin, gentamicin, rifampin, quinupristin/dalfopristin, and daptomycin [6].

Reviewing the effectiveness of daptomycin in treating VRE faecium CNS infections, Lee et al. found that among 10 cases involving daptomycin, 3/10 reported clinical cure with high-dose IV daptomycin (≥8 mg/kg/d) in combination with other agents, 2/10 reported clinical cure with IV daptomycin (6 mg/kg/d) in combination with other agents, and 1/10 reported clinical cure with intraventricular daptomycin and IV linezolid. Among the 4/10 cases with initial treatment failure of IV daptomycin, 3/4 achieved CNS clearance with intraventricular/intrathecal daptomycin, and 1/4 achieved CNS clearance with IV linezolid [6].

Our patient also presented with MDR-*A. baumannii* pneumonia and later developed ventriculitis with a CSF culture growing the same microorganism. The etiology of this latter infection appears to be hematogenous seeding secondary to a transient bacteremia from the initial pneumonia, which has been observed in cases of encephalic aspergillosis [1]. The bacteremia is noted as transient due to negative blood cultures taken five days prior to diagnosing the latter ventriculitis. A case report involving *Enterobacter cloacae* illustrated a clinical scenario similar to ours, with possible hematogenous spread from an initial pneumonia to a subsequent ventriculitis, with sputum and CSF cultures showing the same microorganism while blood cultures yielded no growth [18]. The transient nature of the proposed bacteremia could be explained by the prophylactic systemic antibiotics (PSAs) routinely given throughout the duration of EVDs [19].

Our patient’s MDR-*A. baumannii* ventriculitis was successfully treated with cefiderocol, ampicillin/sulbactam, and intrathecal colistin. Although carbapenems have been the standard for empirical treatment of MDR-*A. baumannii*, the emergence of carbapenem-resistant strains, such as our patient, indicates a need for alternative therapy [12]. Studies have illustrated that colistin is a salvage and life-saving therapy for MDR-*A. baumannii* ventriculitis [8]. Colistin is bactericidal against most gram-negative microorganisms, including *A. baumannii*. However, due to its poor CNS penetration resulting from its high molecular weight and polycationic structure and adverse events related to systemic treatment such as nephrotoxicity and neurotoxicity, colistin is administered via intrathecal/intraventricular (IT/IVT) routes [8,12].

Reviewing the effectiveness of colistin in treating drug-resistant *A. baumannii* CNS infections, Khawcharoneporn et al. performed a combined retrospective study and systematic review and found that among 24 cases involving intrathecal colistin, 10/24 patients received IT/IVT colistin monotherapy and 14/24 patients received combination therapy. No significant differences were found, with 9/10 patients receiving colistin monotherapy reporting clinical cure and 11/14 patients receiving combination therapy involving colistin reporting clinical cure. In addition to colistin, combination therapy included aminoglycosides, imipenem, cefoperazone-sulbactam, ciprofloxacin, and ampicillin-sulbactam [12].

Our patient received a combination therapy of colistin with cefiderocol and ampicillin/sulbactam. Cefiderocol is a novel siderophore-cephalosporin with strong activity against MDR-*A. baumannii* [20]. Susceptibility testing has shown that cefiderocol has significantly lower MIC values than other commonly used Gram-negative agents against drug-resistant-*A. baumannii* [20]. Additionally, time-kill analyses demonstrated synergistic activity with several antibiotics having Gram-negative activity, including ampicillin-sulbactam; the proposed mechanisms of this synergy are increased disruption of the bacterial outer membrane and complementary penicillin-binding protein (PBP) binding [20]. Although cefiderocol monotherapy failed to treat the initial MDR-*A. baumannii* pneumonia, combination therapy with ampicillin-sulbactam and intrathecal colistin successfully treated the subsequent MDR-*A. baumannii* ventriculitis.

## Conclusions

A patient presenting with catheter-associated VRE faecium ventriculitis was successfully treated with linezolid and intrathecal daptomycin. While daptomycin is not approved for Enterococcal infections, the synergistic effect of daptomycin in combination with linezolid proved effective. This patient also presented with MDR-*A. baumannii* pneumonia, with subsequent development of MDR-*A. baumannii* ventriculitis. Although the pneumonia was not cured with cefiderocol monotherapy, the MDR-*A. baumannii* ventriculitis was successfully treated with combination therapy including cefiderocol, ampicillin/sulbactam, and intrathecal colistin. This case report highlights life-saving combination antibiotic therapies for ventriculitis caused by multiple rare and drug-resistant microorganisms.
